# Spatiotemporal denoising of low-dose cardiac CT image sequences using RecycleGAN

**DOI:** 10.1088/2057-1976/acf223

**Published:** 2023-09-12

**Authors:** Shiwei Zhou, Jinyu Yang, Krishnateja Konduri, Junzhou Huang, Lifeng Yu, Mingwu Jin

**Affiliations:** 1 Department of Physics, University of Texas at Arlington, Arlington, TX, United States of America; 2 Department of Computer Science and Engineering, University of Texas at Arlington, Arlington, TX, United States of America; 3 Department of Bioengineering, University of Texas at Arlington, Arlington, TX, United States of America; 4 Department of Radiology, Mayo Clinic, Rochester, MN, United States of America

**Keywords:** CycleGAN, RecycleGAN, denoising, multi-phase CT angiography (MP-CTA)

## Abstract

Electrocardiogram (ECG)-gated multi-phase computed tomography angiography (MP-CTA) is frequently used for diagnosis of coronary artery disease. Radiation dose may become a potential concern as the scan needs to cover a wide range of cardiac phases during a heart cycle. A common method to reduce radiation is to limit the full-dose acquisition to a predefined range of phases while reducing the radiation dose for the rest. Our goal in this study is to develop a spatiotemporal deep learning method to enhance the quality of low-dose CTA images at phases acquired at reduced radiation dose. Recently, we demonstrated that a deep learning method, Cycle-Consistent generative adversarial networks (CycleGAN), could effectively denoise low-dose CT images through spatial image translation without labeled image pairs in both low-dose and full-dose image domains. As CycleGAN does not utilize the temporal information in its denoising mechanism, we propose to use RecycleGAN, which could translate a series of images ordered in time from the low-dose domain to the full-dose domain through an additional recurrent network. To evaluate RecycleGAN, we use the XCAT phantom program, a highly realistic simulation tool based on real patient data, to generate MP-CTA image sequences for 18 patients (14 for training, 2 for validation and 2 for test). Our simulation results show that RecycleGAN can achieve better denoising performance than CycleGAN based on both visual inspection and quantitative metrics. We further demonstrate the superior denoising performance of RecycleGAN using clinical MP-CTA images from 50 patients.

## 1. Introduction

To avoid risks from cardiac catheterization of invasive coronary angiographies (ICAs) in low- and intermediate-risk coronary artery disease (CAD) patients, multi-detector computed tomography (MDCT) has been used for CT angiography (CTA) to noninvasively assess the presence, location, severity, and characteristics of coronary atherosclerosis (Nieman *et al* 2001, Carrigan *et al* 2009, Cademartiri *et al* 2010). In addition, some findings from CTA may not be detectable by ICA (Motoyama *et al* 2009, Sun *et al* 2010, Miszalski-Jamka *et al* 2012). The main challenge in CTA is the strong demand on high temporal resolution (to mitigate cardiac motion artifacts) and high spatial resolution (for small coronary structures), which leads to high radiation dose (Yu *et al* 2009). Electrocardiogram- (ECG-) gated multi-phase CTA (MP-CTA), either in a retrospective helical scan mode or a prospective axial scan mode, can provide much more clinically relevant information than single-phase CTA (SP-CTA). Not only is the important heart function information lost in SP-CTA, but also different parts of the coronary arteries are better seen in different phases (Desjardins and Kazerooni, 2004)_ENREF_49. Thus, MP-CTA may be preferred for much greater diagnostic value than SP-CTA. However, even with ECG tube current modulation (TCM), the average effective dose of a MP-CTA scan could be much higher than 10 mSv (May *et al* 2012)_ENREF_50 (6 ∼ 24 mSv at Mayo Clinic)*,* depending on the width of the pulse window and patient size (Weustink *et al* 2008)_ENREF_51. Taking 80% patients with negative findings into account, minimizing the radiation dose becomes a major and urgent need for a broader application of MP-CTA for CAD diagnosis.

Many methods have been developed to reduce radiation dose in CT acquisition, including optimization of tube current, tube potential, and use of dedicated bowtie filters. However, x-ray dose reduction in general will lead to elevated noise in reconstructed images. The noise in the low-dose CT (LDCT) images can be reduced by either conventional reconstruction methods (La Rivière, 2005, Wang *et al* 2005, Tian *et al* 2011, Wu *et al* 2017, Zhao *et al* 2018, He *et al* 2019, Wu *et al* 2021, Zhou *et al* 2021), or emerging deep-learning based denoising methods directly on images after regular reconstruction through paired-image training (Chen *et al* 2017, Kang *et al* 2017, Wolterink *et al* 2017) or unpaired images training using a cycle-consistent generative adversarial network (CycleGAN) (Kang *et al* 2019, You *et al* 2020, Gu *et al* 2021, Li *et al* 2021). In (Li *et al* 2021), several CycleGAN variants for LDCT denoising were investigated and compared with a paired deep learning method (RED-CNN) (Chen *et al* 2017). However, all these deep learning denoising methods treated each CT image independently and failed to count for the temporal correspondence between images, such as that of MP-CTA image sequences.

To our best knowledge, CycleGAN with an identity loss (Kang *et al* 2019) or wavelet-assisted noise disentanglement (Gu *et al* 2021) was the first work to use deep-learning methods to improve low-dose MP-CTA images. Although CycleGAN can achieve the translation between LDCT and full-dose CT (FDCT) without the need of paired training images, the translation is established only in the spatial domain. The temporal connections among different cardiac phases of a MP-CTA image sequence are not utilized by CycleGAN and may lead to sub-optimal denoising performance. On the other hand, an advanced CycleGAN model with a recurrent loss and a cycle consistency loss over spatial and temporal domain (‘recycle loss’), so called RecycleGAN (Bansal *et al* 2018), was proposed to achieve video-to-video translation in computer vision, which utilizes both spatial and temporal information to solve the translation problem of temporally related data. Nevertheless, RecycleGAN has never been applied to denoise low-dose CT image sequences including MP-CTA. In this work, we adapt RecycleGAN to take into consideration of the temporal connection of the succeeding cardiac phases of MP-CTA images. This novel deep learning denoising method not only enjoys the advantage of CycleGAN without need of paired training images, but also exploits both spatial and temporal correspondence to boost denoising performance for time series of MP-CTA images. As our aim in this work focuses on comparing the denoising performance of CycleGAN and RecycleGAN for low-dose MP-CTA images, the comparison between CycleGAN and other traditional and deep learning methods for LDCT denoising can be found in the previous works, such as (Li *et al* 2021).

## 2. Methods

### 2.1. CycleGAN

**Figure 1.**
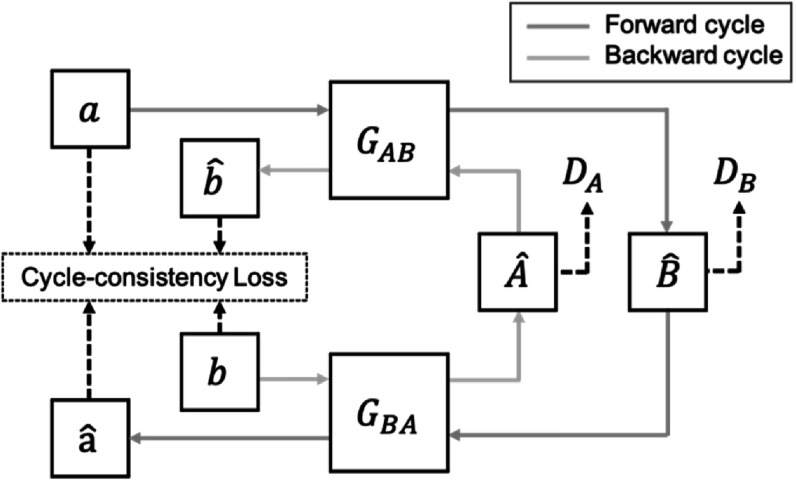
The workflow of CycleGAN. In the forward cycle (blue line), an image $a$ from domain $A$ is translated to domain $B$ by generator ${G}_{{AB}},$ expressed as ${\hat{B}=G}_{{AB}}\left(a\right).$ Then, $\hat{B}$ is translated back to domain $A,$ expressed as $\hat{a}={G}_{{BA}}\left({G}_{{AB}}\left(a\right)\right).$ The backward cycle (green line) has similar operations where image $b$ in domain $B$ is mapped to domain $A$ as ${\hat{A}=G}_{{BA}}\left(b\right)$ and then mapped back to domain $B$ as ${\hat{b}=G}_{{AB}}\left({G}_{{BA}}\left(b\right)\right).$

To achieve image-to-image translation, CycleGAN (Zhu *et al* 2017) is proposed to learn mapping functions between two different domains without the need of paired data. Formally, given a set of images from a source domain $A$ (e.g., low-dose CT images) and a set of images from a target domain $B$ (e.g., full-dose CT images), the goal of CycleGAN is to learn a mapping ${G}_{{AB}}:A\longrightarrow B,$ such that the output ${G}_{{AB}}\left(a\right)$ is indistinguishable from the images in domain $B.$ The architecture of CycleGAN is composed of two generators and two adversarial discriminators (figure 1). Specifically, each generator aims to translate images from one domain to the other domain, while each discriminator is designed to distinguish between the real images in the target domain and the translated images from the source domain.

The objective of CycleGAN contains two terms: an adversarial loss (Goodfellow *et al* 2014) and a cycle consistency loss (Zhu *et al* 2017). Given data distribution $a\sim {p}_{{data}}\left(a\right)$ and $b\sim {p}_{{data}}\left(b\right),$ the adversarial loss is designed to match the distribution of generated images ${G}_{{AB}}\left(a\right)$ to the distribution of that in the target domain $B$ as follows:

\begin{eqnarray*}\begin{array}{c}{{\mathscr{L}}}_{{GAN}}\left({G}_{{AB}},{D}_{B},A,B\right)\\ =\,{{\mathbb{E}}}_{b\unicode{x0007E}{P}_{{data}}\left(b\right)}\left[{\mathrm{log}}{D}_{B}\left(b\right)\right]\\ +{{\mathbb{E}}}_{a\unicode{x0007E}{P}_{{data}}\left(a\right)}\left[{\mathrm{log}}\left(1-{D}_{B}\left({G}_{{AB}}\left(a\right)\right)\right)\right]\end{array},\end{eqnarray*}

where ${G}_{{AB}}$ aims to minimize this loss against an adversary ${D}_{B}$ that tries to maximize it, i.e., $\mathop{{\mathrm{\min }}}\limits_{{G}_{{AB}}}\mathop{{\mathrm{\max }}}\limits_{{D}_{B}}{{\mathscr{L}}}_{{GAN}}\left({G}_{{AB}},{D}_{B},A,B\right)$ (Zhu *et al* 2017). Similarly, for the generator ${G}_{{BA}},$ the adversarial loss is:

\begin{eqnarray*}\begin{array}{c}{{\mathscr{L}}}_{{GAN}}\left({G}_{{BA}},{D}_{A},A,B\right)\\ =\,{{\mathbb{E}}}_{a\unicode{x0007E}{P}_{{data}}\left(a\right)}\left[{\mathrm{log}}{D}_{A}\left(a\right)\right]\\ +{{\mathbb{E}}}_{b\unicode{x0007E}{P}_{{data}}\left(b\right)}\left[{\mathrm{log}}\left(1-{D}_{A}\left({G}_{{BA}}\left(b\right)\right)\right)\right]\end{array}\end{eqnarray*}

To further reduce the space of possible mapping functions (Goodfellow *et al* 2014), a cycle-consistency loss is introduced to guarantee the output of each cycle to be close to the input to that cycle, i.e., ${G}_{{BA}}\left({G}_{{AB}}\left(a\right)\right)\approx a$ and ${G}_{{AB}}\left({G}_{{BA}}\left(b\right)\right)\approx b.$ The cycle-consistency loss is defined as:

\begin{eqnarray*}\begin{array}{c}{{\mathscr{L}}}_{{cycle}}\left({G}_{{AB}},{G}_{{BA}}\right)\\ =\,{{\mathbb{E}}}_{a\unicode{x0007E}{P}_{{data}}\left(a\right)}\left[{\unicode{x02016}{G}_{{BA}}\left({G}_{{AB}}\left(a\right)\right)-a\unicode{x02016}}_{1}\right]\\ +{{\mathbb{E}}}_{b\unicode{x0007E}{P}_{{data}}\left(b\right)}\left[{\unicode{x02016}{G}_{{AB}}\left({G}_{{BA}}\left(b\right)\right)-b\unicode{x02016}}_{1}\right]\end{array}\end{eqnarray*}

This cycle-consistency loss enforces the constraint that ${G}_{{AB}}$ and ${G}_{{BA}}$ be inverse of each other (Kang *et al* 2019).

By putting all losses together, the overall objective for CycleGAN is:

\begin{eqnarray*}\begin{array}{c}{{\mathscr{L}}}_{{cycleGAN}}\left({G}_{{AB}},{G}_{{BA}},{D}_{A},{D}_{b}\right)\\ =\,{{\mathscr{L}}}_{{GAN}}\left({G}_{{AB}},{D}_{B},A,B\right)+{{\mathscr{L}}}_{{GAN}}\\ \times \left({G}_{{BA}},{D}_{A},B,A\right)+\lambda {{\mathscr{L}}}_{{cycle}}\left({G}_{{AB}},{G}_{{BA}}\right)\end{array},\end{eqnarray*}

where $\lambda $ controls the importance between the adversarial losses and the cycle-consistency loss.

The variants of CycleGAN (Zhu *et al* 2017) have been applied to various domains (Li *et al* 2021). However, they only use the spatial information in 2D images, and do not use the temporal information for the optimization of the image translation model (Bansal *et al* 2018).

The cycle-consistency loss forces the optimization to learn a solution that is closely tied to the input. This is suitable for the situation that only spatial information is available during the translation, while for time-related image sequences, such as CTA images, with only the cycle consistency, the model may be inadequate to generate perceptually unique results. The network structure of CycleGAN used in this work is based on figures 4 and 5 in (Li *et al* 2021).

### 2.2. RecycleGAN

**Figure 2.**
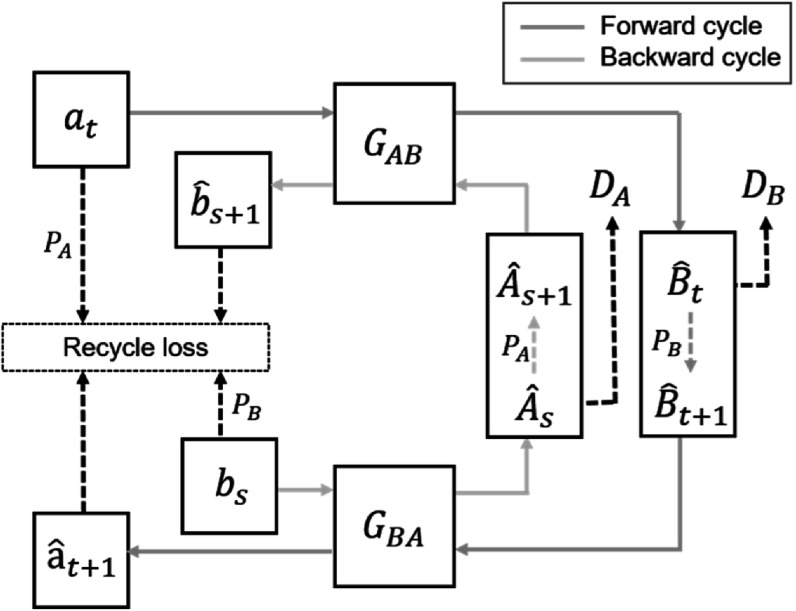
The workflow of RecycleGAN. In the forward cycle (blue line), an image ${a}_{t}$ at time $t$ from domain $A$ is translated to domain $B$ by generator ${G}_{{AB}},$ expressed as ${\hat{{B}_{t}}=G}_{{AB}}\left({a}_{t}\right).$ Then, a temporal predictor ${P}_{B}$ is applied on $\hat{{B}_{1:t}}$ to predict a future image ${\hat{B}}_{t+1},$ and then ${\hat{B}}_{t+1}$ is translated back to domain $A,$ expressed as $\hat{{a}_{t+1}}={G}_{{BA}}\left({{P}_{B}(G}_{{AB}}\left({a}_{t}\right))\right).$ The backward cycle (green line) has similar operations where image ${b}_{s}$ in domain $B$ is mapped to domain $A$ as ${\hat{{A}_{s}}=G}_{{BA}}\left({b}_{s}\right),$ and then mapped back to domain $B$ with a temporal predictor ${P}_{A},$ expressed as ${\hat{{b}_{s+1}}=G}_{{AB}}\left({{P}_{A}(G}_{{BA}}\left({b}_{s}\right))\right).$

RecycleGAN (Bansal *et al* 2018) is proposed to learn a mapping between two videos from different domains. It utilizes both spatial and temporal information to solve the reconstruction problem of temporally related data. RecycleGAN shares a similar model framework with CycleGAN, except that the cycle-consistency loss is replaced by a recurrent loss and a recycle loss to make use of the temporally ordered images to learn a better mapping. The workflow of RecycleGAN is shown in figure 2.

Given unpaired but ordered images $\left({a}_{1},{a}_{2},\ldots ,{a}_{t},\ldots \right)\in A$ (i.e., temporally ordered low-dose MP-CTA images) and $\left({b}_{1},{b}_{2},\ldots ,{b}_{s},\ldots \right)\in B$ (i.e., temporally ordered full-dose MP-CTA images), a recurrent temporal predictor ${P}_{A}$ is trained to predict the future image given the past images. The recurrent loss is defined as:

\begin{eqnarray*}{{\mathscr{L}}}_{\tau }\left({P}_{A}\right)=\displaystyle \sum _{t}{\unicode{x02016}{a}_{t+1}-{P}_{A}\left({a}_{1:t}\right)\unicode{x02016}}^{2},\end{eqnarray*}

where ${a}_{1:t}=\left({a}_{1},\ldots ,{a}_{t}\right).$ Then the recycle loss across domains can be defined based on this temporal prediction model as follows:

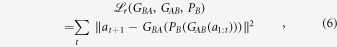

where ${G}_{{AB}}\left({a}_{1:t}\right)=\left({G}_{{AB}}\left({a}_{1}\right),{G}_{{AB}}\left({a}_{2}\right),\ldots ,{G}_{{AB}}\left({a}_{t}\right)\right).$ In both forward and backward cycles, the recycle loss requires a sequence of images to map back to the initial domain. The overall loss of ReCycleGAN is defined by:

\begin{eqnarray*}\begin{array}{c}{ {\mathcal L} }_{recycleGAN}\left(G,P,D\right)={ {\mathcal L} }_{GAN}\left({G}_{AB},{D}_{B},A,B\right)\\ +{ {\mathcal L} }_{GAN}\left({G}_{BA},{D}_{A},B,A\right)+\,{\lambda }_{rx}{ {\mathcal L} }_{r}\left({G}_{BA},{G}_{AB},{P}_{B}\right){\mathrm{}}\\ +{\lambda }_{ry}{ {\mathcal L} }_{r}\left({G}_{AB},{G}_{BA},{P}_{A}\right)+\,{\lambda }_{\tau x}{ {\mathcal L} }_{\tau }\left({P}_{A}\right)\,+{\lambda }_{\tau y}{ {\mathcal L} }_{\tau }\left({P}_{B}\right),\end{array}\end{eqnarray*}

where $\lambda $’s control the importance of the losses. We show in the experiments that the proposed method provides an effective translation from low-dose MP-CTA to full-dose MP-CTA images when learning from unpaired CT image sequences. The detailed network structure of RecycleGAN (Bansal *et al* 2018) can be found in appendix.

## 3. Experimental setting

### 3.1. Phantom data

We used the XCAT phantom program (Segars *et al* 2010) based on 18 patients’ data (nine females and nine males) to generate cardiac CT images (thorax 512 × 512 × 128, voxel size of 1 mm^3^) for two different dose levels: full-dose and low-dose (20% of the full-dose). The number of phases for each cardiac cycle is set to eight. The 18 phantoms were divided into nine pairs of female and male. To generalize the performance of CycleGAN and RecycleGAN, we used the 9-fold cross-validation (CV). For each CV, the training dataset contains seven pairs of female and male phantoms, the validation dataset contains a pair of female and male phantoms, and the testing dataset contains another pair of female and male phantoms. The table 1 shows the patient pairs for nine CV sets. For each CV, the network was trained using the training data and the hyperparameters were tuned using the validation data. Afterward, the optimal hyperparameters were used to train the network using both training and validation datasets. Finally, the denoising performance was evaluated on the test dataset. To account for the temporal relationship among cardiac phases, the images of each slice are viewed as a looped video of eight image frames.

**Table 1.** The cross-validation (CV) settings.

CV number	Training patients	Validation patients	Testing patients
1	#2, #3, #4, #5, #6, #7, #9	#8	#1
2	#1, #3, #4, #5, #6, #7, #8	#9	#2
3	#1, #4, #5, #6, #7, #8, #9	#2	#3
4	#1, #2, #3, #5, #7, #8, #9	#6	#4
5	#1, #2, #4, #6, #7, #8, #9	#3	#5
6	#1, #2, #3, #4, #5, #8, #9	#7	#6
7	#1, #2, #3, #5, #6, #8, #9	#4	#7
8	#2, #3, #4, #5, #6, #7, #9	#1	#8
9	#1, #2, #3, #4, #6, #7, #8	#5	#9

### 3.2. Patient data

We also used the real patient MP-CTA images from Mayo Clinic to evaluate the performance of RecycleGAN. MP-CTA images of 50 patients were retrospectively collected and deidentified (IRB was approved by Mayo Clinic). Intravenous iodinated contrast (Omnipaque^®^ 350) was injected using a bolus tracking technique, where the volume and injection rate were determined by the patient weight, followed by 10 c.c. saline chaser. The arterial attenuation enhancement is 200~350 HU. These cases were acquired using a routine retrospectively ECG-gated helical scanning technique on a 3rd generation 192-slice dual-source scanner (Force, Siemens Healthcare): 0.25 sec rotation time, 192 × 0.6 mm detector configuration, helical pitch automatically selected based on heart rate, tube potential automatically determined (CAREkV), TCM (CAREDose4D, maximum tube current (MTC) 180 mAs in the pulse window and 20% outside), and ECG-pulsing at 40%-70% phases. These parameters may vary for some patients, especially for those with irregular heartbeat. The CTDIvol was varying from patient to patient depending on the patient size, heart rate, and regularity of the heart rate (31∼120 mGy, i.e. 6∼24 mSv). For irregular heart rate, the pulsing window may be extended automatically, which could dramatically increase radiation dose. 3D volume images (512×512 in plane, 300~375 slices, isotropic 0.4 mm size) at 20 phases (0%-95% windows) were reconstructed using the Siemens ADMIRE algorithm with a Qr40 kernel (ADMIRE strength setting of 3). Therefore, in 20 phases of CTA images of each patient, roughly 6 phases are of full dose (with MTC) while the remaining 14 phases are of low dose (with 20% MTC). Due to the patient size, heartbeat irregularity, and unbalanced full-dose and low-dose slices (# of full-dose slices ≪ # of low-dose slices), we selected the full-dose slices and the low-dose slices for training (48 patients out of 50) based on the standard deviation (STD) of a square region in the aorta (full dose <39 HU and low dose >59 HU) and at least three consecutive phases falling into either the full-dose window or the low-dose window. To keep the underlying data similar, we selected 16.2 thousand low-dose images and 15.8 thousand full-dose images for CycleGAN training, while we selected 15.8 thousand low-dose frames and 15.2 thousand full-dose frames for RecycleGAN training. The difference was caused by the requirement of three consecutive phases for RecycleGAN training, which was not satisfied by all CycleGAN training images. To tune the model hyperparameters, MP-CTA images of one patient were used for the validation set. The remaining one patient dataset was served as the test set for performance evaluation.

### 3.3. Evaluation metrics

To evaluate the proposed method, peak signal-to-noise ratio (PSNR) (Huynh-Thu and Ghanbari, 2008) and structural similarity index (SSIM) (Zhou *et al* 2004, Horé and Ziou, 2010) are used as quantitative measurements for the XCAT phantom data. The PSNR is an expression for the ratio between the (denoised) low-dose CT image $x$ and the corresponding full-dose CT image $y$ as follows,

\begin{eqnarray*}{PSNR}=10{{\mathrm{log}}}_{10}\left(\frac{{MA}{X}_{Y}^{2}}{{MSE}}\right),\end{eqnarray*}

where ${MA}{X}_{Y}$ is the maximum signal value that is set as 4095 for 12-bit CT images in our experiments. The term ‘MSE’ stands for mean squared error and is defined as,

\begin{eqnarray*}\begin{array}{c}{MSE}=\frac{1}{{mn}}\displaystyle \sum _{i=0}^{m-1}\displaystyle \sum _{j=0}^{n-1}{\left[x\left(i,j\right)-y\left(i,j\right)\right]}^{2}\end{array}\,,\end{eqnarray*}

where $i$ and $j$ are the row and column indices of low-dose CT image $x$ and the corresponding full-dose CT image $y,$ respectively, and $m$ and $n$ represent the number of rows the number of columns, respectively. The PSNR measures the cumulative difference between two images. The higher the PSNR, the better the performance of denoising.

In addition to PSNR, the SSIM is designed to compare luminance, contrast, and structure difference between two images and is defined as,

\begin{eqnarray*}\begin{array}{c}{SSIM}\left(x,y\right)=l\left(x,y\right)c\left(x,y\right)s\left(x,y\right)\end{array},\end{eqnarray*}

where $l\left(x,y\right)=\frac{2{\mu }_{x}{\mu }_{y}+{c}_{1}}{{\mu }_{x}^{2}+{\mu }_{y}^{2}+{c}_{1}},$ $c\left(x,y\right)=\frac{2{\sigma }_{{xy}}+{c}_{2}}{{\sigma }_{x}^{2}+{\sigma }_{y}^{2}+{c}_{2}},$ and $s\left(x,y\right)=\frac{{\sigma }_{{xy}}+{c}_{3}}{{\sigma }_{x}{\sigma }_{y}+{c}_{3}}.$ The first term $l\left(x,y\right)$ measures closeness of mean luminance ${\mu }_{x}$ and ${\mu }_{y}.$ The contrast $c\left(x,y\right)$ is measured by standard deviation ${\sigma }_{x}$ and ${\sigma }_{y}.$ The structure similarity $s\left(x,y\right)$ is measured by correlation coefficient between images $x$ and $y.$ ${\sigma }_{{xy}}$ is the covariance between two images. The ${c}_{1},$ ${c}_{2}$ and ${c}_{3}$ are used to stabilize the division operation (Zhou *et al* 2004, Horé and Ziou, 2010) . The higher SSIM value indicates the closer resemblance of two images.

For patient data, since the ground truth was unknown, the performance was evaluated using STD in a square region of the aorta of CTA images of the test patient, where the uniform intensity is expected. Therefore, the lower STD, the better denoising performance.

### 3.4. Hyperparameters

Hyperparameters of CycleGAN and RecycleGAN were generally kept the same as the previous publications(Bansal *et al* 2018, Li *et al* 2021). Specifically, for CycleGAN $\lambda $ was set to 10, while for RecycleGAN, ${\lambda }_{rx}$ was set to 0.5, and ${\lambda }_{{ry}}$ was set to 50, ${\lambda }_{\tau x}$ was set to 1, and ${\lambda }_{\tau y}$ was set to 100. The networks were trained with random weights from scratch using the Adam solver. For each model, we searched for the best learning rate in the range of 5.00 × 10^−6^ to 1.26 × 10^−3^ based on the lowest PSNR of the validation set (a pair of female and male patients for the phantom data and one patient for the patient data). For the phantom data, after training each CV data set, the best performing model was applied on the test dataset for performance evaluation. For the patient data, the learning rate was tuned using the validation patient and the best model was applied on the test patient.

## 4. Results

### 4.1. Phantom results

**Figure 3.**
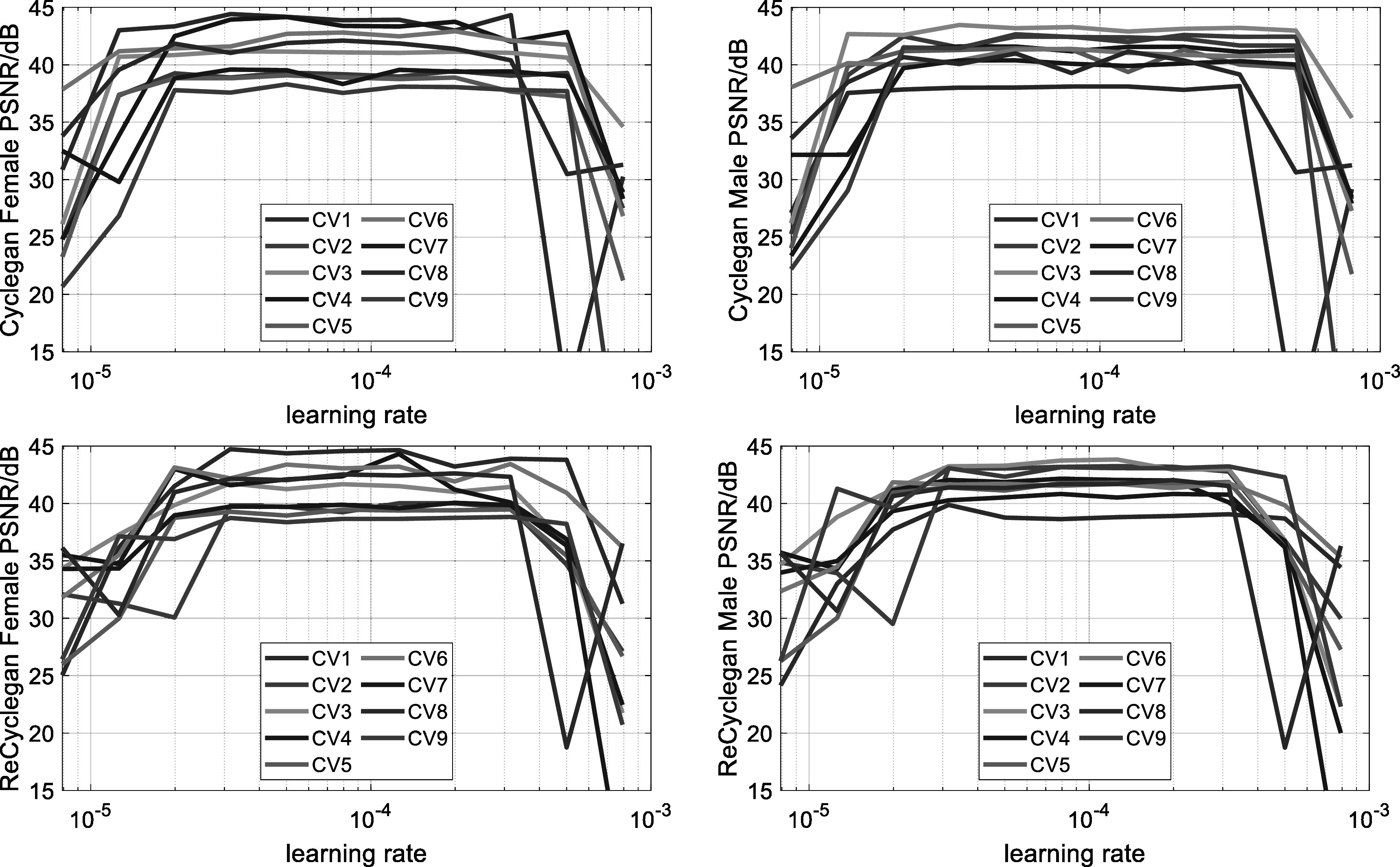
The validate PSNR changes for CycleGAN and RecycleGAN with different learning rates. (Top row: CycleGAN; Bottom row: RecycleGAN. Left column: female; Right column: male).

We compared our proposed spatiotemporal RecycleGAN method with CycleGAN using PSNR and SSIM as quantitative metrics. Figure 3. shows PSNR changes of the validation set along with different learning rates for nine CV sets. We separated the female and male validation PSNR as some large differences were found between the genders (see tables 2 and 3). For CycleGAN, the learning rates 2 × 10^−5^ to 3 × 10^−4^ seem to have a PSNR plateau for the validation set. For RecycleGAN, this range narrows to 3 × 10^−5^ to 3 × 10^−4^. The best validation PSNR for each CV set was listed in table 2 along with SSIM. First, the different PSNR and SSIM performance can be clearly seen between female and male validation patients. In most cases for CycleGAN, the PSNR differences are 2–6 dB except for CV7 (less than 1 dB), while SSIM difference is ranged from more than 0.01 to about 0.07. This difference is mainly caused by the learning rate was tuned based on the overall PSNR using both female and male validation patients. Although the differences are also observed for RecycleGAN metrics, they are notably smaller. RecycleGAN outperformances CycleGAN in almost all cases, except for CV7 male SSIM (marked as bold blue in table 2). After taking the average values (± Standard Deviation) of nine CV sets, the PSNR and SSIM for CycleGAN are 41.23 ± 2.16 dB and 0.9462 ± 0.0241 for the female validation data, and 41.13 ± 1.62 dB and 0.9526 ± 0.0188 for the male validation data. The corresponding numbers for RecycleGAN are 41.71 ± 2.07 dB and 0.9523 ± 0.0224 for the female validation data, and 42.10 ± 1.17 dB and 0.9600 ± 0.0108 for the male validation data. RecycleGAN achieves not only the greater average values, but also the smaller variances than CycleGAN. The best models were then applied to the test dataset and the PSNR and SSIM results are shown in table 3. The similar findings to the best validation metrics are observed although the number of cases that RecycleGAN is worse than CycleGAN increases from one to two. RecycleGAN still outperformances CycleGAN in most cases, except for PSNR of CV1 male and CV8 female (marked as bold blue in table 3). The PSNR and SSIM for CycleGAN are 40.36 ± 2.23 dB and 0.9431 ± 0.0250 for the female test data, and 40.91 ± 2.16 dB and 0.9501 ± 0.0208 for the male test data. The corresponding numbers for RecycleGAN are 40.84 ± 2.05 dB and 0.9512 ± 0.0215 for the female test data, and 41.43 ± 2.11 dB and 0.9572 ± 0.0178 for the male test data. The test results demonstrated again that RecycleGAN leads to better denoising performance than CycleGAN.

**Table 2.** The best validation metrics for CycleGAN and RecycleGAN.

	CycleGAN				RecycleGAN			
Cross-Validation	Female PSNR	Female SSIM	Male PSNR	Male SSIM	Female PSNR	Female SSIM	Male PSNR	Male SSIM
1	44.42	0.9761	38.01	0.9071	44.73	0.9814	39.88	0.9394
2	39.32	0.9284	42.46	0.9658	40.02	0.9367	43.25	0.9710
3	41.17	0.9494	43.47	0.9729	41.75	0.9575	43.83	0.9745
4	44.16	0.9762	41.59	0.9547	44.32	0.9778	42.05	0.9592
5	39.08	0.9258	41.45	0.9552	39.48	0.9303	41.87	0.9578
6	42.92	0.9679	40.86	0.9549	43.44	0.9723	41.77	0.9603
7	39.59	0.9265	40.38	0.9609	40.06	0.9335	40.83	0.9458
8	42.11	0.9600	39.28	0.9359	42.62	0.9640	42.04	0.9611
9	38.30	0.9060	42.66	0.9654	38.83	0.9153	43.23	0.9696
Average	41.23	0.9462	41.13	0.9526	41.71	0.9523	42.10	0.9600
Standard Deviation	2.16	0.0241	1.62	0.0188	2.07	0.0224	1.17	0.0108

**Table 3.** Quantitative metrics for the test data for CycleGAN and RecycleGAN.

	CycleGAN				RecycleGAN			
Cross-Validation	Female PSNR	Female SSIM	Male PSNR	Male SSIM	Female PSNR	Female SSIM	Male PSNR	Male SSIM
1	42.18	0.9601	41.31	0.9562	42.33	0.9665	41.28	0.9620
2	41.03	0.9467	43.26	0.9706	41.70	0.9533	43.83	0.9749
3	39.04	0.9245	41.44	0.9543	40.40	0.9474	42.67	0.9677
4	39.54	0.9261	40.34	0.9408	40.00	0.9321	40.71	0.9452
5	37.92	0.8993	42.64	0.9645	38.74	0.9116	42.93	0.9687
6	37.40	0.9636	37.16	0.9396	37.55	0.9668	37.60	0.9495
7	43.16	0.9680	41.36	0.9547	43.62	0.9711	41.96	0.9594
8	43.57	0.9721	37.77	0.9026	43.29	0.9761	38.71	0.9174
9	39.44	0.9274	42.90	0.9672	39.94	0.9358	43.19	0.9702
Average	40.36	0.9431	40.91	0.9501	40.84	0.9512	41.43	0.9572
Standard Deviation	2.23	0.0250	2.16	0.0208	2.05	0.0215	2.11	0.0178

**Figure 4.**
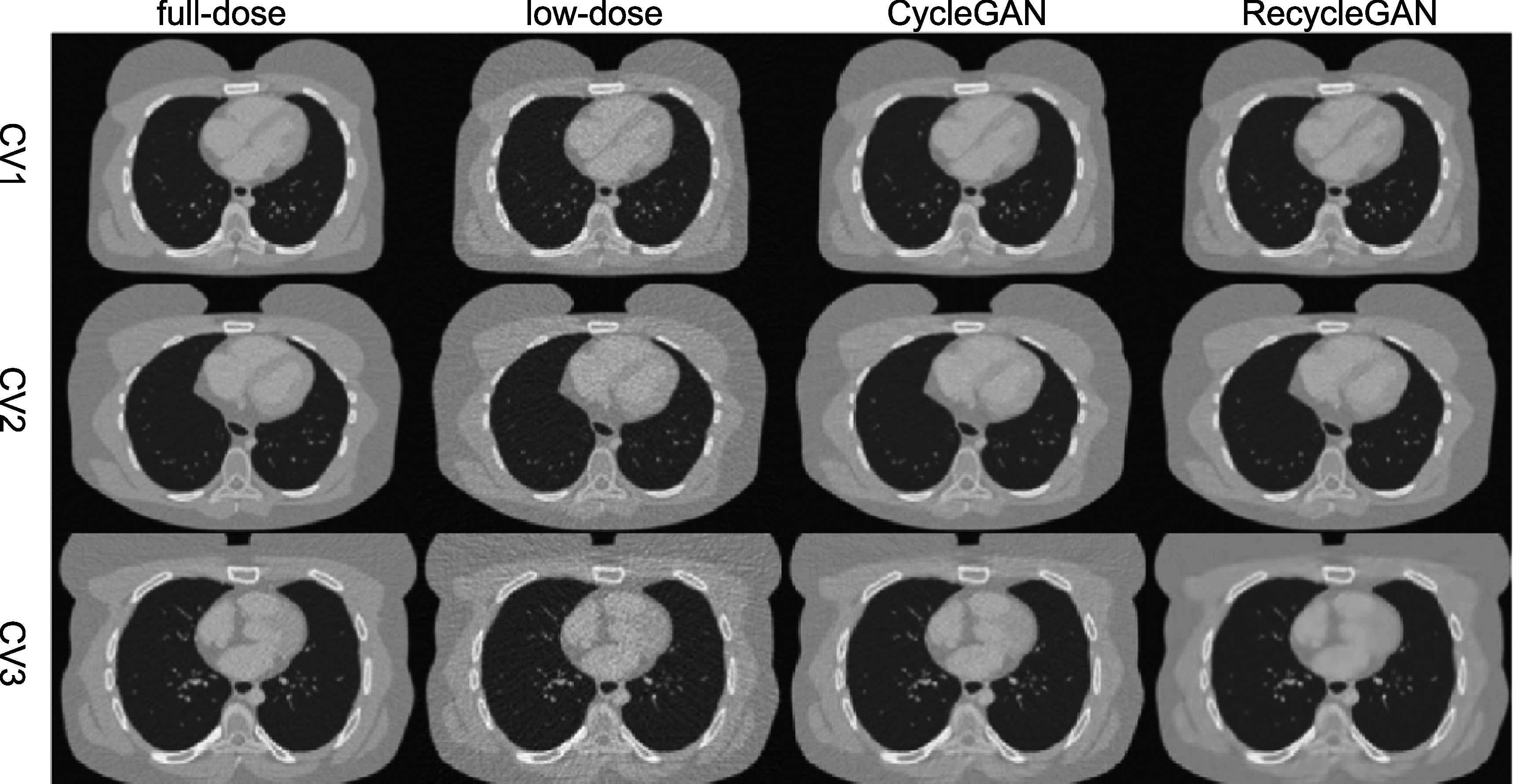
The transverse slice of phase 5 in the testing dataset for female (Display window [−1000, 550]HU).

**Figure 5.**
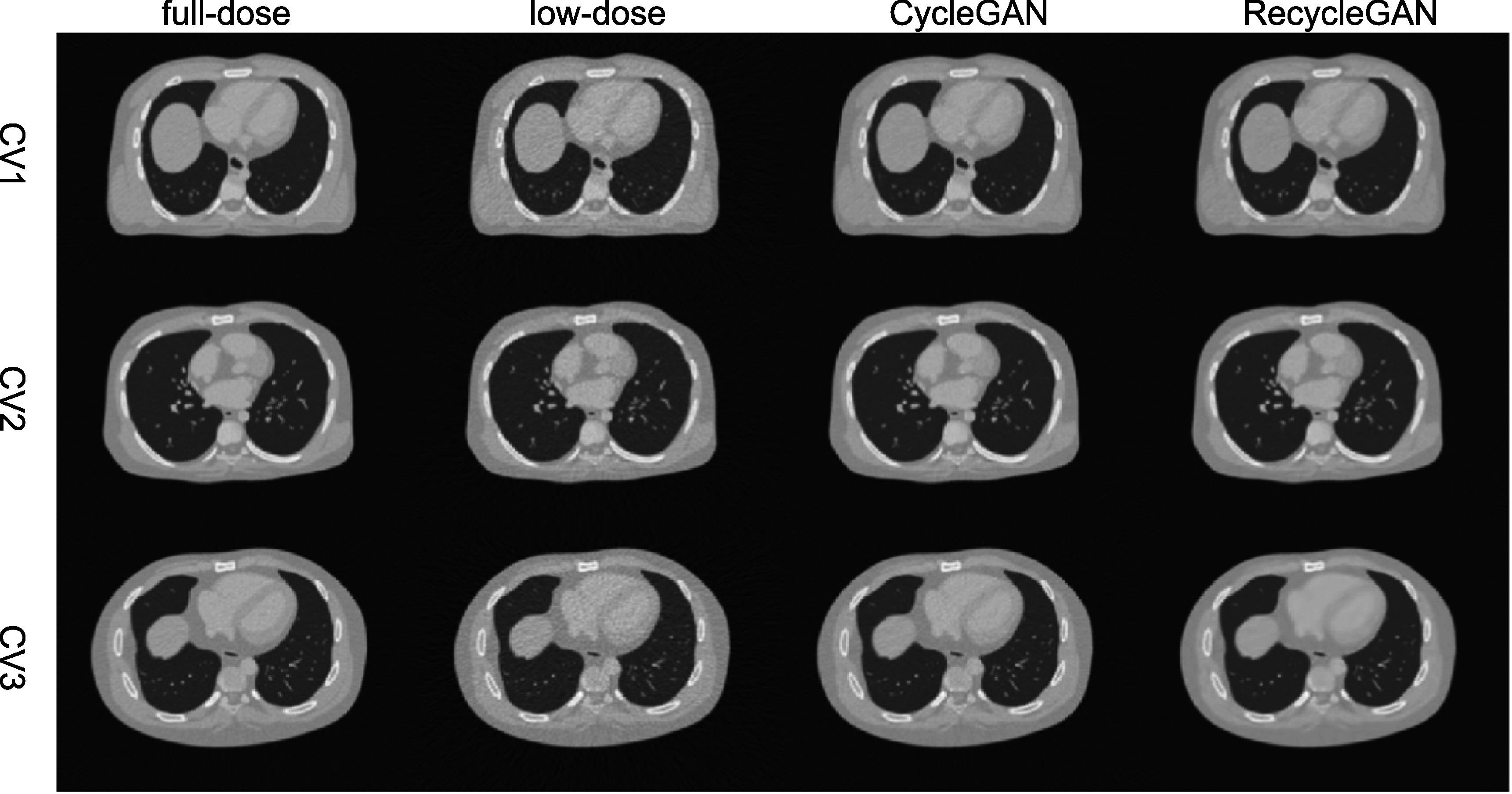
The transverse slice of phase 5 in the testing dataset for male (Display window [−1000, 550]HU).

We show an image (Phase 5) of the test female and male data denoised by CycleGAN and RecycleGAN for CV1, CV2 and CV3 in figures 4 (female) and 5 (male), respectively. The full-dose and low-dose images are also shown as reference. Both CycleGAN and RecycleGAN effectively remove the noise in the images. RecycleGAN has less noise and is closer to the full-dose images than CycleGAN as shown in figures 4 and 5.

**Figure 6.**
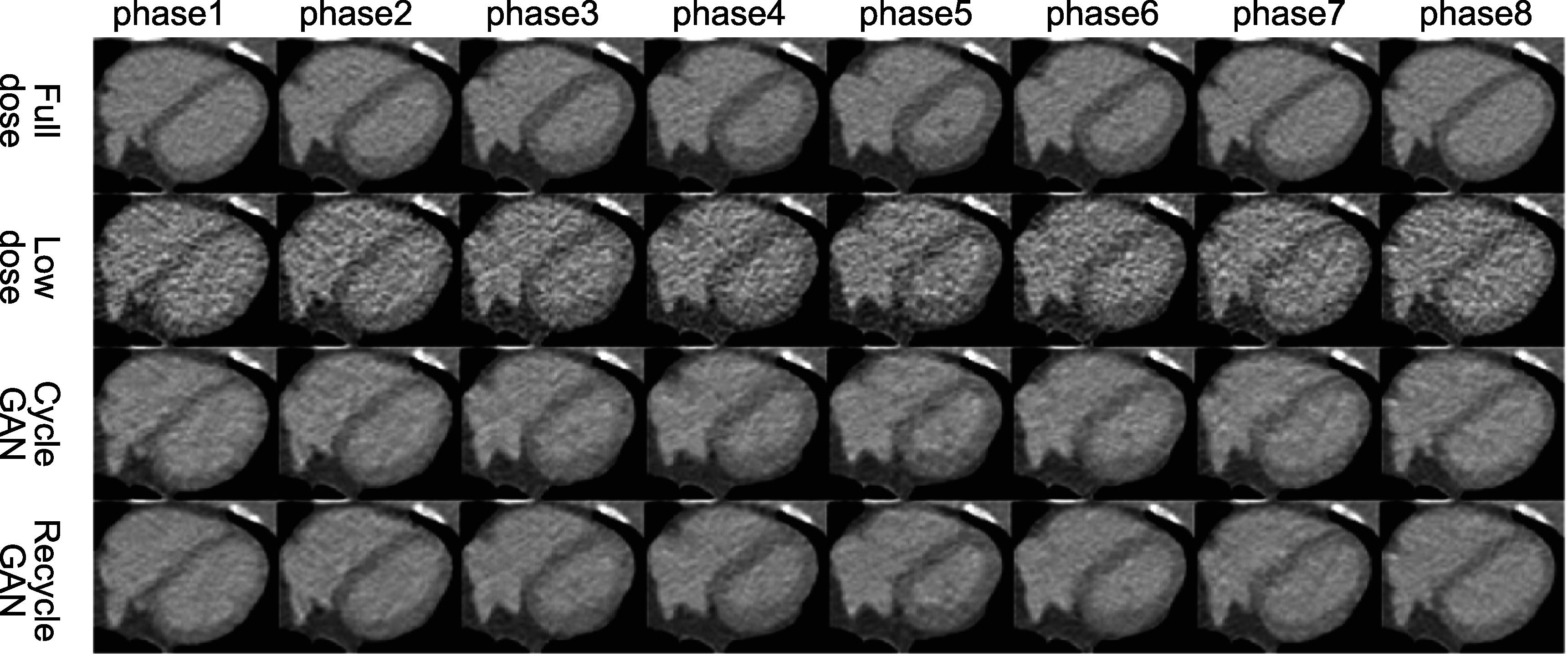
Eight phases of the heart region for CV2 female test data (Display Window [−215 335]HU).

**Figure 7.**
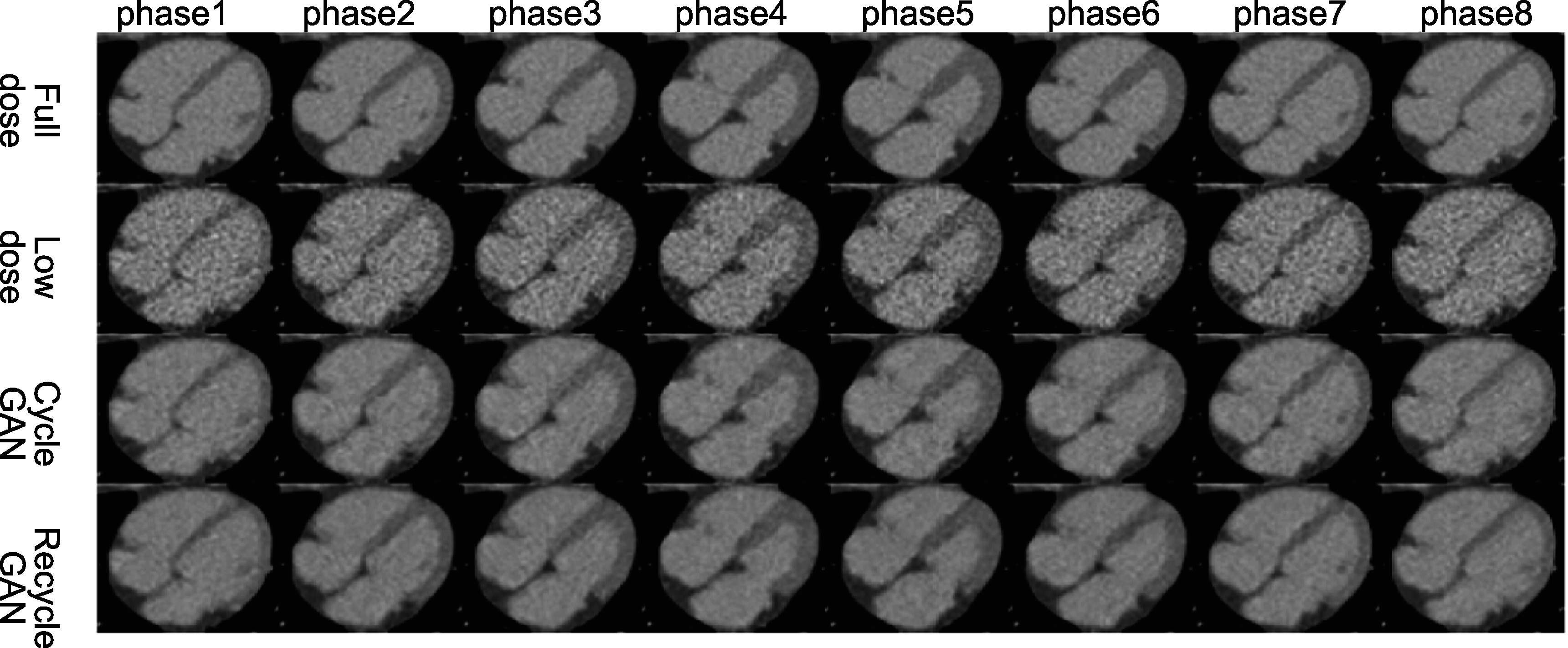
Eight phases of the heart region for CV2 male test data (Display Window [−215 335]HU).

In figures 6 and 7, the eight phases of the heart region are shown for different methods along with the full-dose and low-dose references. Again, both CycleGAN and RecycleGAN effectively suppress the noise. RecycleGAN does a better job to further remove the noise than CycleGAN in the myocardium and the blood pool. RecycleGAN also achieves better contrast and structure preservation than CycleGAN.

### 4.2. Patient results

**Figure 8.**
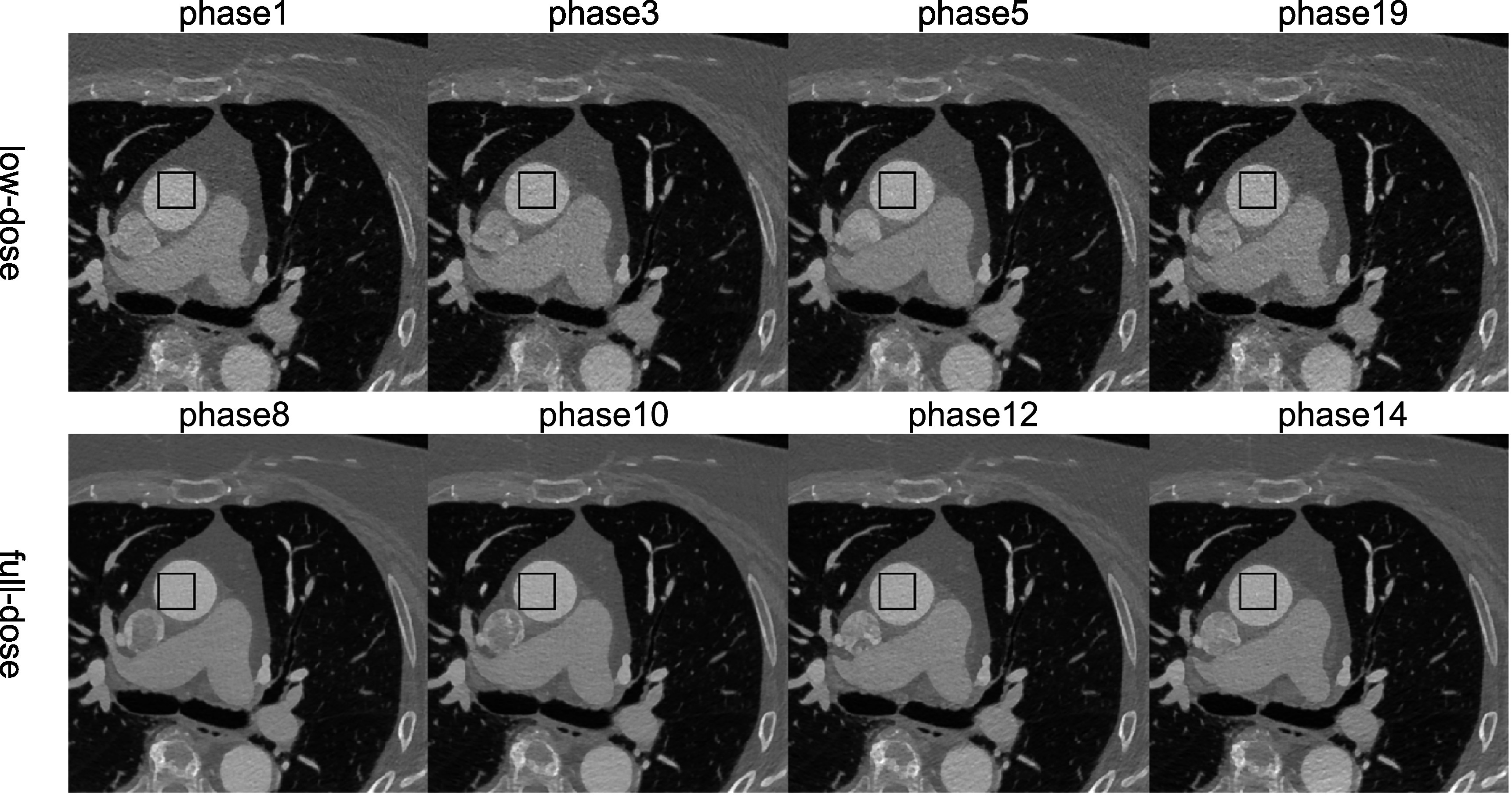
Twenty phases of the test patient CTA images (Display Window [−1000 950]HU). The black box in the aorta is used as a region of interest (ROI) to calculate the standard deviation (STD) of the intensity to represent the noise level.

**Figure 9.**
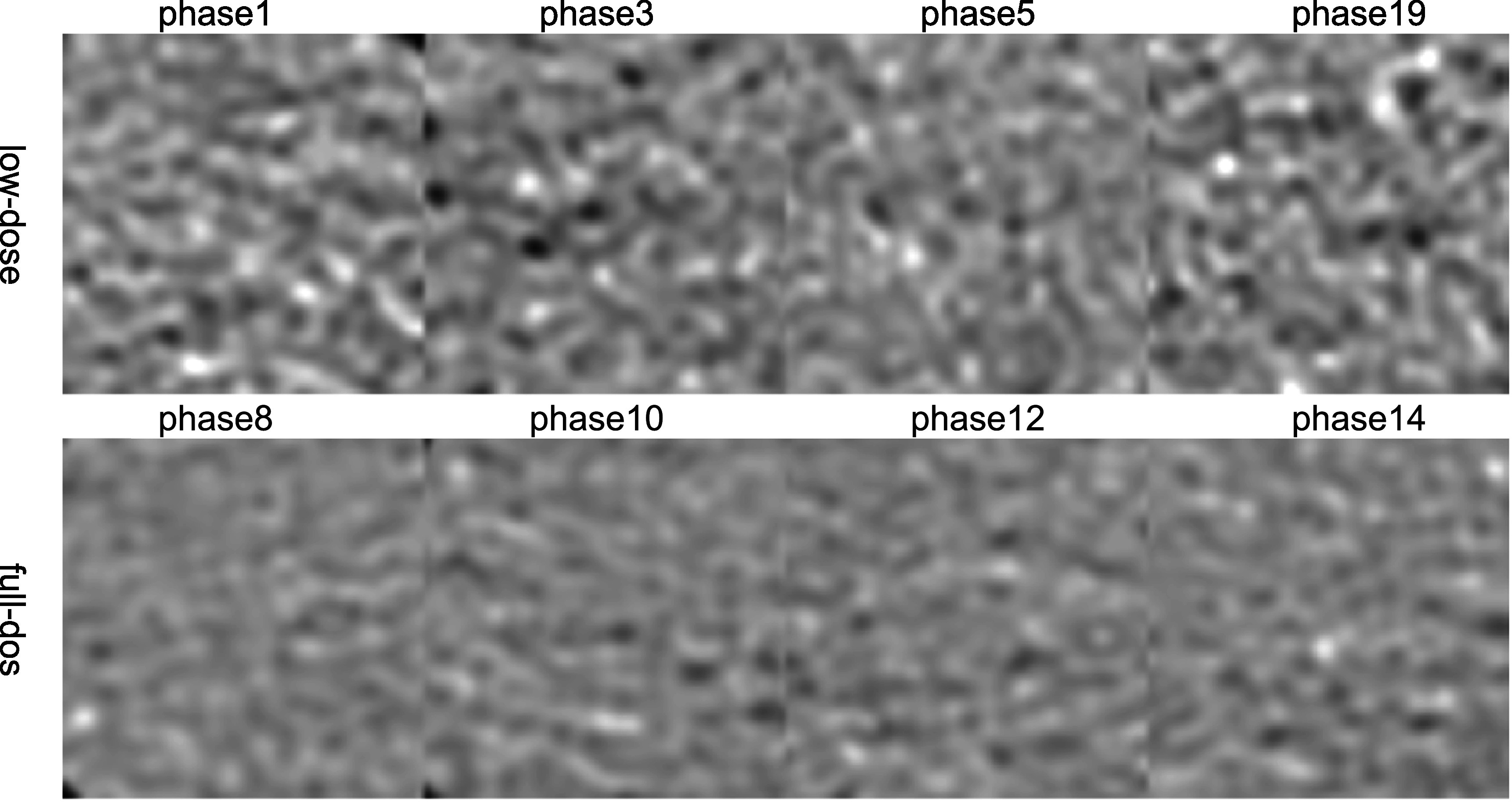
Twenty phases of the ROI (black box in figure 10) (Display Window [76 676]HU).

For the patient CTA data, some phases are with full-dose (at 100% MTC) and some with low-dose (at 20% MTC or transition between 100% MTC to 20% MTC). Phase 8–13 in this test patient should be in the 100% MTC window (full dose), while others should be in the 20% MTC window (low dose) or the transition window. For clarity, four phases for each category are shown in figure 8, i.e. phases 1, 3, 5, and 19 for low-dose with high noise and phases 8, 10, 12, and 14 for full-dose with low noise. Note that phase 5 shows less noise than other low-dose phases as the MTC was ramped up during phases 4–6. The black box in the aorta in figure 8 is used as region of interest (ROI) to calculate the standard deviation (STD) of the intensity to represent the noise level and the magnified views of ROI are shown in figure 9. The noise texture can be seen more clearly, and the top row (low-dose images) are much noisier than the bottom row (full-dose images). The STD values in HU for eight low-dose phases and eight high-dose phases are listed in table 4, where the low-dose STD values are greater than 45 HU and the full-dose STD values are less than 40. It is worth noting that they are different from the thresholds for the selection of low-dose and full-dose training data (full dose <39 HU and low dose >59 HU). The inclusion of transition phases (4–6) is to see how effective CycleGAN and ReCycleGAN can denoise for different noise levels in the test data.

**Table 4.** The standard deviation (STD) values in HU of low-dose and full-dose ROI.

Phase	1	2	3	4	5	6	19	20
Low-dose	60.12	62.35	58.17	49.34	45.06	51.09	65.61	66.56
phase	8	9	10	11	12	13	14	15
Full-dose	31.09	29.01	36.84	34.17	35.27	35.33	38.39	39.59

**Figure 10.**
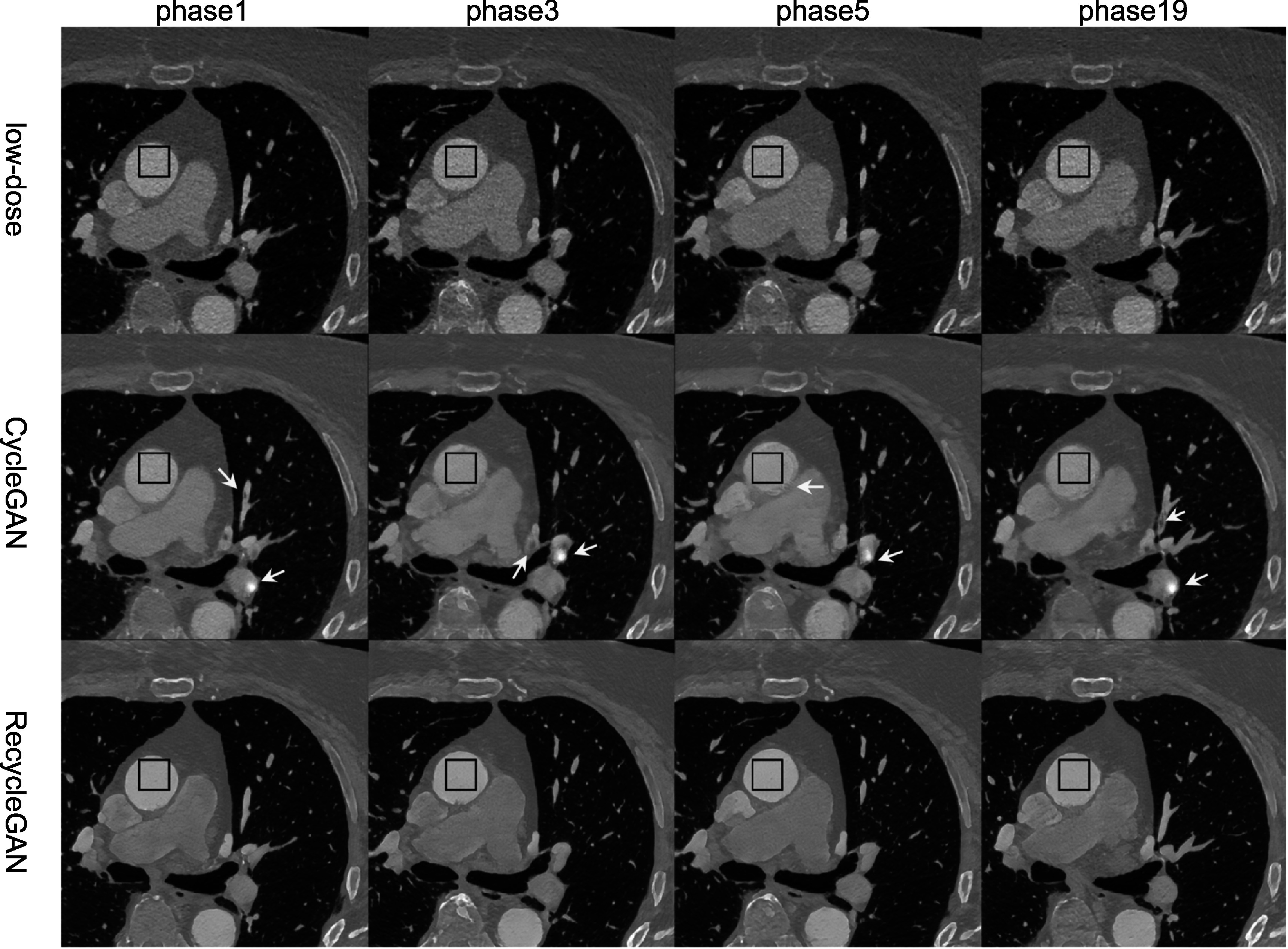
Low-dose phases of the test patient denoised by different methods. (Top row: original low-dose images; middle row: CycleGAN; bottom row: RecycleGAN) Display Window [−1000 950]HU.

**Figure 11.**
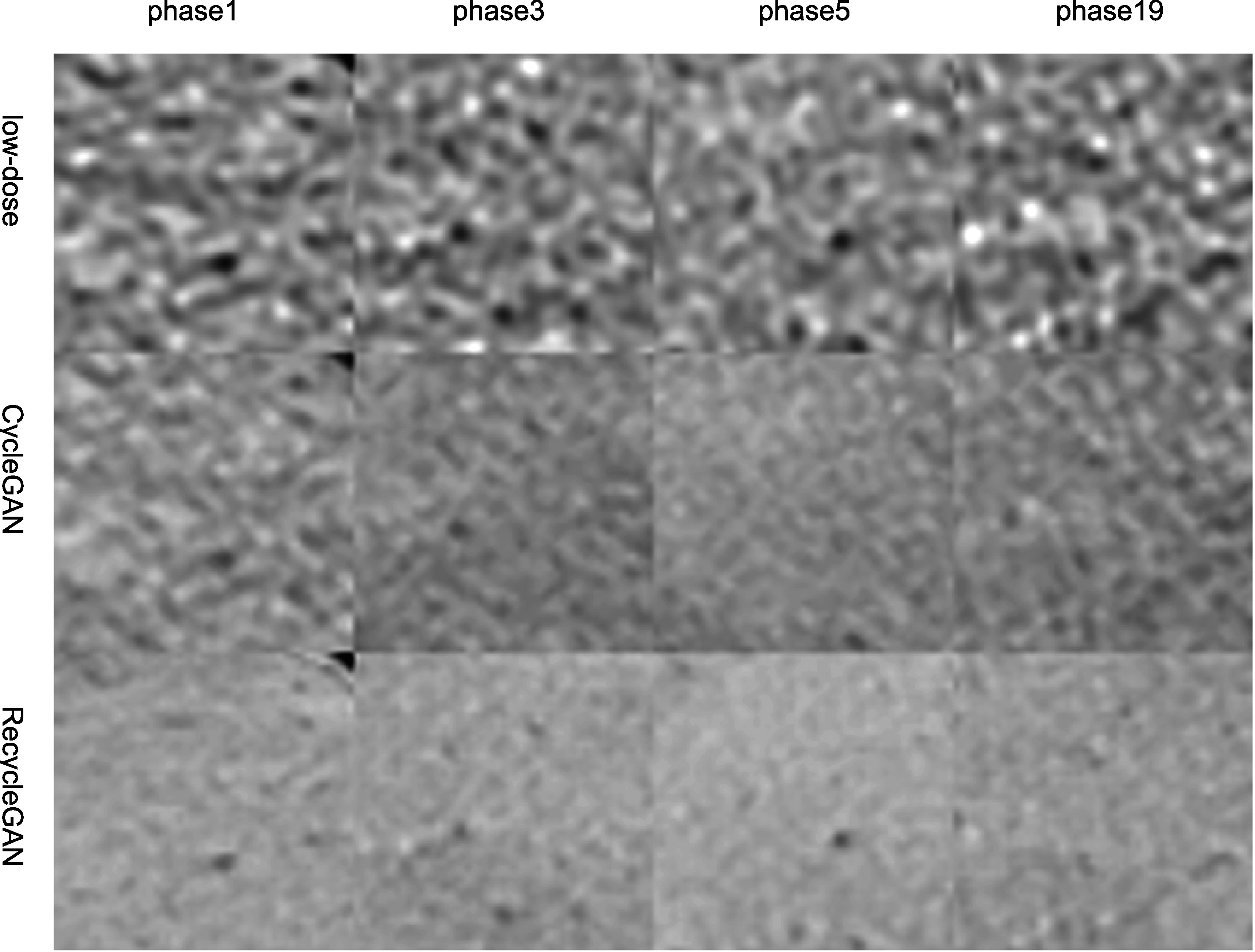
Images of the test patient in a region of interest denoised by different methods. (Top row: original low-dose images; middle row: CycleGAN; bottom row: RecycleGAN) Display Window [76 676]HU.

In figure 10, we compared the low-dose CTA images (phases 1, 3, 5, and 19) of the test patient with CycleGAN and RecycleGAN denoised images. Similar to the findings in the phantom results, both CycleGAN and RecycleGAN can effectively suppress the noise, while RecycleGAN keeps the image details much better than CycleGAN. CycleGAN also suffers from some intensity artifacts as marked by the yellow arrows in figure 10, which are consistent with those reported in the previous study (Gu *et al* 2021). The ROI images are shown in figure 11, CycleGAN and RecycleGAN yield lower noise compared to the original low-dose images. Furthermore, RecycleGAN images are least noisy and more consistent across all phases, while CycleGAN suffers some artificial pattens and noise bumps for phase 5. The consistency of RecycleGAN is likely due to the recurrent loss, which takes the temporal correlation into the denoising mechanism. The quantitative measures of STD of ROI for eight phases of low-dose and high-dose phases are shown in table 5. CycleGAN does a good job for most phases (bringing down the noise from 50~60 HU to 30~40 HU) except for phase 6. RecycleGAN further suppresses the noise to the range of 16~26 HU for all phases.

**Table 5.** The standard deviation (STD) values in HU in ROI for CycleGAN and RecycleGAN.

Phase	1	2	3	4	5	6	19	20
Low-dose	60.12	62.35	58.17	49.34	45.06	51.09	65.61	66.56
CycleGAN	40.46	36.48	34.50	30.43	38.87	54.59	53.14	46.46
RecycleGAN	26.01	26.70	23.75	19.82	17.16	23.30	20.48	25.32

## 5. Discussion and conclusions

RecycleGAN is more effective than CycleGAN for denoising low-dose CT image sequences as it uses a recurrent loss to enforce the temporal consistence. In essence, it treats 2D image series as a 3D signal (2D space + 1D time) and denoises in 3D instead of 2D. This leads to more effective and consistent noise suppression and structure preservation. In the future, the whole 3D volume image plus time may be treated as a 4D signal to see if further improvement could be achieved. Right now, the training of RecycleGAN is more time consuming (37 h for RecycleGAN versus 18 h for CycleGAN for the phantom data on NVIDIA A6000 GPU). The computational burden moving from 3D to 4D may be alleviated by multiple GPU parallelism.

In this work, we focus on comparing RecycleGAN and CycleGAN with extensive phantom and patient studies (with 9-fold cross-validation for the phantom study and 50 patients for the patient study). We used CycleGAN as a baseline, which was extensively compared with other state-of-the-art denoising methods (You *et al* 2020, Li *et al* 2021). Although the direct comparison between RecycleGAN and other methods may be lack in this work, their relative performance can be deduced from the comparison between RecycleGAN and CycleGAN.

MP-CTA can offer more diagnostic information than SP-CTA. However, the full radiation dose is a major hurdle to adopt MP-CTA broadly for CAD diagnosis. Therefore, to lower MP-CTA dose level to be comparable to SP-CTA will be clinically significant. RecycleGAN is an important development moving toward this goal. First, RecycleGAN is a software-based method and does not require the aligned low-dose and full-dose images. Although the hardware difference may demand further tuning of the RecycleGAN model trained on a certain type of scanner (e.g. Siemens Force in this work), as the nature of CT images is the same, a comprehensive model could be built using data from multi-scanners and multi-centers. Secondly, RecycleGAN showed superior performance on suppressing noise and preserving the structure details and contrast for CTA image sequences compared to CycleGAN. If a constant 20% MTC could be used for MP-CTA, the radiation dose could be lowered by ~55% (assuming 6 phases 100%MTC pulse window for a total of 20 phases). Although this dose level is still higher than SP-CTA, further reduction, such as sparse sampling, could be exploited. Use of advance deep learning or reconstruction methods to explore the lower bound of MP-CTA dose level without compromising the diagnostic outcomes is worth further investigation.

For the patient MP-CTA cases used in this study, an ECG-gated tube current modulation was turned on with the pulsing window between 40% and 70% of the cardiac phases. The tube current reduction outside the pulsing window was 20% of the full tube current. Therefore, this study focused on reducing noise of low-dose images acquired outside the pulsing window. One previous study has investigated CycleGAN denoising of extreme low-dose (high-noise) CT (Gu *et al*, 2021). At 4% of full dose, although the baseline CycleGAN method(Kang *et al* 2019) introduces some artificial features, CycleGAN denoised images still improved the signal-to-noise ratio (SNR) and the radiologist reading rates over the original LDCT images. To address the performance deterioration of CycleGAN, the wavelet-assisted noise disentanglement (WAND) (Gu *et al* 2021) was introduced to extract high-frequency sub-band images (including both noise and edge information) before CycleGAN training. Their results showed that WAND were effective to suppress high noise and avoid artifacts. In figure 10, we also discovered similar artifacts in CycleGAN images reported in (Gu et al, 2021), which were successfully removed in RecycleGAN images. This demonstrated that the spatiotemporal training in RecycleGAN may be an alternative way to correct for the inconsistent translation of CycleGAN. Nevertheless, we believe that WAND can be deployed similarly to RecycleGAN, i.e. adding high-frequency sub-band image extraction before RecycleGAN training, when its denoising performance is significantly degraded due to substantially elevated noise. This will be a topic for future investigation.

In summary, we developed a spatiotemporal deep learning denoising method, RecycleGAN, for low-dose cardiac CT image sequences. Compared to the state-of-the-art spatial domain denoising method, CycleGAN, RecycleGAN utilizes the temporal relationship of several consecutive phases through a recurrent loss to further improve the denoising performance. Note that RecycleGAN still enjoys the advantage of CycleGAN without need of aligned low-noise and high-noise images. Both phantom and patient studies show that RecycleGAN outperforms CycleGAN in quantitative metrics and image quality for CT image sequences. It is envisioned that RecycleGAN could be used to significantly lower the MP-CTA imaging dose by effectively removing the image noise. More clinically relevant evaluations will be conducted in the future work.

## Data Availability

The data cannot be made publicly available upon publication because they contain sensitive personal information. The data that support the findings of this study are available upon reasonable request from the authors.

## Appendix

### The network structure of RecycleGAN

The generator, predictor, and discriminator of RecycleGAN are shown in tables A1, A2, and A3, respectively (Bansal *et al* 2018).

**Table A1.** The generator structure of RecycleGAN.

Function blocks (level # - layer #)	Kernel Shape
Generator	—
├─Sequential: 1-1	—
│ └─ReflectionPad2d: 2-1	—
│ └─Conv2d: 2-2	[1, 64, 7, 7]
│ └─InstanceNorm2d: 2–3	—
│ └─ReLU: 2–4	—
│ └─Conv2d: 2–5	[64, 128, 3, 3]
│ └─InstanceNorm2d: 2–6	—
│ └─ReLU: 2–7	—
│ └─Conv2d: 2–8	[128, 256, 3, 3]
│ └─InstanceNorm2d: 2–9	—
│ └─ReLU: 2–10	—
│ └─ResnetBlock: 2–11	—
│ └─ResnetBlock: 2–12	—
│ └─ResnetBlock: 2–13	—
│ └─ResnetBlock: 2–14	—
│ └─ResnetBlock: 2–15	—
│ └─ResnetBlock: 2–16	—
│ └─ConvTranspose2d: 2–17	[128, 256, 3, 3]
│ └─InstanceNorm2d: 2–18	—
│ └─ReLU: 2–19	—
│ └─ConvTranspose2d: 2–20	[64, 128, 3, 3]
│ └─InstanceNorm2d: 2–21	—
│ └─ReLU: 2–22	—
│ └─ReflectionPad2d: 2–23	—
│ └─Conv2d: 2–24	[64, 1, 7, 7]
│ └─Tanh: 2–25	—

**Table A2.** The predictor structure of RecycleGAN.

**Table A3.** The discriminator structure of RecycleGAN.

Function blocks (level # - layer #)	Kernel Shape
Discriminator	—
├─Sequential: 1-1	—
│ └─Conv2d: 2-1	[1, 64, 4, 4]
│ └─LeakyReLU: 2-2	—
│ └─Conv2d: 2–3	[64, 128, 4, 4]
│ └─InstanceNorm2d: 2–4	—
│ └─LeakyReLU: 2–5	—
│ └─Conv2d: 2–6	[128, 256, 4, 4]
│ └─InstanceNorm2d: 2–7	—
│ └─LeakyReLU: 2–8	—
│ └─Conv2d: 2–9	[256, 512, 4, 4]
│ └─InstanceNorm2d: 2–10	—
│ └─LeakyReLU: 2–11	—
│ └─Conv2d: 2–12	[512, 1, 4, 4]

Definition of fundamental blocks: Conv2d: 2D convolution ReLU: rectified linear unit function LeakyReLU: A type of activation function based on a ReLU, which has a small slope for negative values instead of strict zero ConvTranspose2d: Applies a 2D transposed convolution operator over an input image composed of several input planes. Tanh: Applies the Hyperbolic Tangent function element-wise. ReflectionPad2d: Pads the input tensor using the reflection of the input boundary. InstanceNorm2d: Applies Instance Normalization over a 4D input ResnetBlock: Sequence of ReflectionPad2d, Conv2d(shape of [256, 256, 3, 3]), InstanceNorm2d, ReLU, ReflectionPad2d, Conv2d(shape of [256, 256, 3, 3]), and InstanceNorm2d functions
